# Genetic characterization of *Cryptosporidium* spp. in Hotan Black Chickens in China reveals two novel subtypes of *Cryptosporidium meleagridis*[Fn FN1]

**DOI:** 10.1051/parasite/2022051

**Published:** 2022-11-09

**Authors:** Xinwei Feng, Luyao Xin, Fuchang Yu, Xianming Song, Jianing Zhang, Jinhua Deng, Meng Qi, Wei Zhao

**Affiliations:** 1 College of Animal Science and Technology, Tarim University Alar Xinjiang 843300 China; 2 Xinjiang Agricultural Vocational Technical College Changji Xinjiang 831199 China; 3 Department of Parasitology, Wenzhou Medical University Wenzhou Zhejiang 325035 China

**Keywords:** *Cryptosporidium*, Genetic, Subtype, Chicken, China

## Abstract

A total of 617 fecal specimens were collected on 18 Hotan Black chicken farms in Southern Xinjiang, China, and tested for the presence of *Cryptosporidium* spp. by PCR of the small subunit ribosomal RNA (*SSU* rRNA) gene. The overall infection rate by *Cryptosporidium* spp. was 11.5% (71/617), and ten of the 18 farms were positive. The infection rate by *Cryptosporidium* spp. was 14.5% (48/331) in the 30–60 d group, higher than chickens in the <30 d (12.0%, 15/125), 60–90 d (6.9%, 5/72), and >90 d (3.4%, 3/89) groups. *Cryptosporidium meleagridis* (*n* = 38) and *C. baileyi* (*n* = 33) were confirmed by sequencing analysis. A total of 25 of the 38 *C. meleagridis-*positive specimens were subtyped successfully at the *gp60* gene, including one known subtype (IIIbA23G1R1, *n* = 1) and two novel subtypes, named IIIbA25G1R1 (*n* = 20) and IIIbA31G1R1 (*n* = 4). The results showed that infection by *Cryptosporidium* spp. in Hotan Black Chickens was common in this area and the distribution of *C. meleagridis* subtypes had regional characteristics.

## Abstract (fr)

Un total de 617 échantillons fécaux ont été prélevés dans 18 élevages de poulets noirs du Hotan dans le sud du Xinjiang, en Chine, et testés pour la présence de *Cryptosporidium* spp. par PCR du gène de la petite sous-unité de l’ARN ribosomique (ARNr SSU). Le taux d’infection global par *Cryptosporidium* spp. était de 11,5 % (71/617) et dix des 18 élevages étaient positifs. Le taux d’infection de *Cryptosporidium* spp. était de 14,5 % (48/331) dans le groupe 30–60 jours, supérieur à celui des poulets dans les groupes <30 jours (12,0 %, 15/125), 60–90 jours (6,9 %, 5/72) et >90 jours (3,4 %, 3/89). *Cryptosporidium meleagridis* (*n* = 38) et *C. baileyi* (*n* = 33) ont été confirmés par analyse de séquençage. Vingt-cinq des 38 spécimens positifs pour *C. meleagridis* ont été sous-typés avec succès au niveau du gène gp60, dont un sous-type connu (IIIbA23G1R1, *n* = 1) et deux nouveaux sous-types, nommés IIIbA25G1R1 (*n* = 20) et IIIbA31G1R1 (*n* = 4). Les résultats ont montré que l’infection par *Cryptosporidium* spp. chez les poulets noirs du Hotan était commune dans cette zone et que la distribution des sous-types de *C. meleagridis* avait des caractéristiques régionales.

## Introduction

*Cryptosporidium* is considered an important zoonotic protozoan parasite of various animals, including mammals, birds, reptiles, fishes, and amphibians [10]. Morphological, biological, and molecular data have validated 46 recognized *Cryptosporidium* species [15, 24]. Among these, 23 *Cryptosporidium* species have been identified in humans [23]. Epidemiological data have revealed that most human cryptosporidiosis cases are caused by *C. hominis*, *C. parvum*, and *C. meleagridis* [23]. DNA sequence analysis of the 60-kDa glycoprotein (*gp60*) gene is the most common subtyping tool [34]. In previous subtyping studies of *C. meleagridis* based on the *gp60* gene, ten subtype families (IIIa to IIIj) have been established, and eight (IIIa to IIIh) of which were found in birds and humans [23]. Based on the principle of “One Health”, it is a key step to prevent and control cryptosporidiosis by investigating infection by *Cryptosporidium* in animal hosts at the subtype level.

Chickens are among the most widespread domestic animals. Currently, seven species (*C. meleagridis*, *C. baileyi*, *C. parvum, C. galli, C. andersoni*, *C. ubiquitum* and *C. ornithophilus*) and five genotypes (*C. avian* genotypes VII, VIII, IX, *C. genotype* BrPR1 and *C. xiaoi*-like genotype) of *Cryptosporidium* have been recognized in chickens [26]. In China, the infection rate by *Cryptosporidium* in chickens was reported to be 1.3% to 10.2% in Shandong, Henan, Hebei, Hubei and Zhejiang Provinces, with *C. baileyi* being the dominant *Cryptosporidium* species [4, 11, 21, 32, 33]. Hotan Black chicken is an excellent local breed, mainly distributed in the Minfeng, Lop, and Hotan counties of the Xinjiang Uygur Autonomous Region (herein referred to as Xinjiang), China. However, the prevalence and species/subtypes of *Cryptosporidium* in Hotan Black chicken remain unclear. This study aimed to examine the occurrence and genetic characterization of *Cryptosporidium* in Hotan Black chickens in Xinjiang.

## Materials and methods

### Ethics statement

The Animal Ethical Committee of Wenzhou Medical University (WMU-2020-12) reviewed and approved the protocol of this study. Before beginning the study, we contacted the owners of these animals and obtained their permission to profile their chickens. Fecal specimens were collected without harming the animals.

### Specimens collection

**Figure 1:**
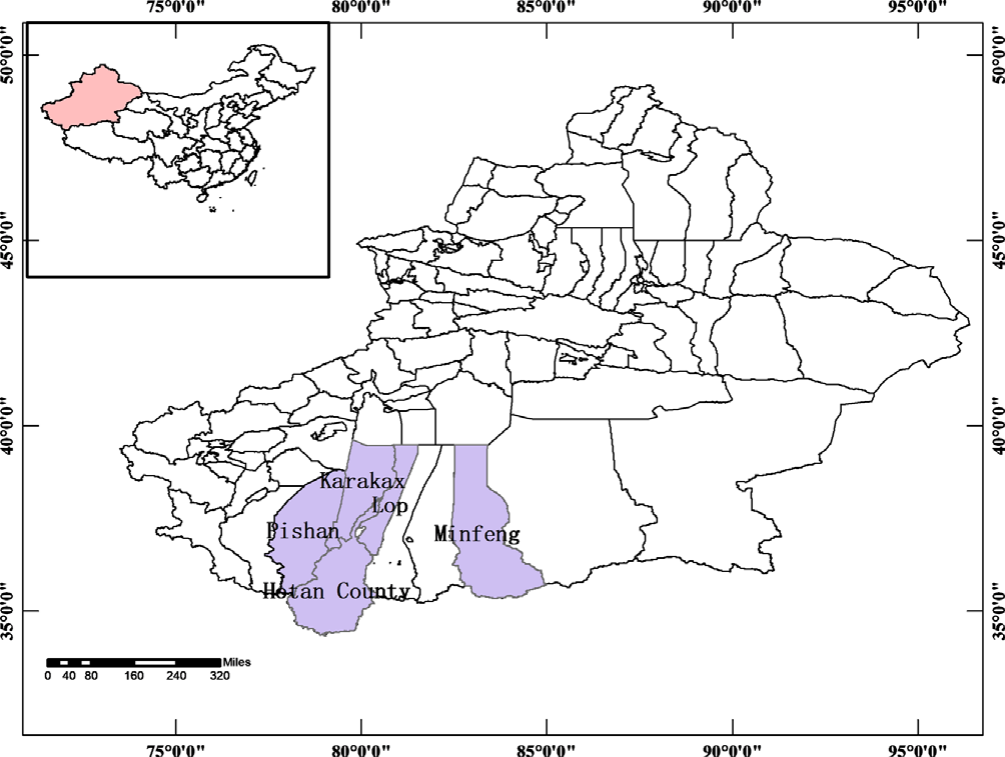
Specific locations where specimens were collected in this study.

Between June 2020 and July 2021, 617 chickens were collected from 18 private farms in villages of Hotan, Lop, Minfeng, Karakax, and Pishan counties of Xinjiang (Fig. 1). The farm owners randomly selected the chickens that were subsequently housed in individual flat-surfaced cages away from contact with other animals. Then, one fecal specimen (2–5 g) for each animal was collected from the tray under the individual cage using a sterile disposable latex glove and placed into an individual sterile plastic container. All the specimens were kept in iceboxes, transported to the laboratory and stored at 4 °C. All chickens were healthy looking without any apparent infection at sampling.

### DNA extraction

Approximately 200 mg of stool specimens were picked using autoclaved disposable bamboo sticks to extract genomic DNA of *Cryptosporidium* using an E.Z.N.A. Stool DNA kit (Omega Biotek Inc., Norcross, GA, USA), following the manufacturer’s procedure. The eluted DNA (200 μL) was kept at −20 °C until PCR analysis.

### Genotyping and subtyping of *Cryptosporidium* spp.

All extracted DNA were examined by nested PCR at the *SSU* rRNA gene of *Cryptosporidium* [35]. *Cryptosporidium-*positive specimens were subtyped by nested PCR at the *gp60* gene [31]. EasyTaq PCR SuperMix (TransGene Biotech Co. Ltd., Beijing, China) was used for all PCR amplifications. Double distilled water and cattle derived *C. andersoni* DNA were used as the negative and positive control, respectively. Each specimen was PCR amplified twice. All the secondary PCR products were resolved on 1.5% agarose gels stained with GelRed (Biotium Inc., Fremont, CA, USA).

### Nucleotide sequence analysis

All positive secondary PCR products were sent for bidirectional sequencing at GENEWIZ (Suzhou, China). The obtained sequences were aligned against each other and reference sequences from GenBank using the Basic Local Alignment Search Tool (BLAST) (http://blast.ncbi.nlm.nih.gov/Blast.cgi) and Clustal X 2.1 (http://www.clustal.org/).

### Phylogenetic analyses

A phylogenetic tree was constructed in MEGA7 using the maximum likelihood method based on the Tamura-Nei model to evaluate the genetic relationship between the *gp60* subtypes of *C. meleagridis*. The representative nucleotide sequences obtained in this study were deposited in the GenBank database under accession numbers: ON209450 to ON209452 for the *SSU* rRNA gene and ON210984 to ON210986 for the *gp*60 gene.

### Statistical analysis

A chi-square analysis determined the correlation between the *Cryptosporidium* prevalence in farm regions and age groups using SPSS version 19.0. Results with *p* < 0.05 were considered statistically significant.

## Results

### Occurrence of *Cryptosporidium* in Hotan Black chickens

The overall infection rate by *Cryptosporidium* in Hotan Black chickens was 11.5% (71/617). Ten out of the 18 farms had *Cryptosporidium* positive specimens, with the highest infection rate in Farm Hotan5 (46.2%, 6/13), and the lowest in farm Pishan18 (2.4%, 1/41) (Table 1). The infection rates by *Cryptosporidium* in chickens on each farm were significantly different (*χ*^2^ = 142.2, *df* = 17, *p* < 0.01). The *Cryptosporidium* infection rate in the 30–60 d Hotan Black chickens (14.5%, 48/331) was higher than in the <30 d (12.0%, 15/125), 60–90 d (6.9%, 5/72), and >90 d (3.4%, 3/89) groups (Table 2), with significant differences among different age groups (*χ*^2^ = 10.202, *df* = 3, *p* < 0.05).

**Table 1:** Prevalence and distribution of *C. baileyi* and *C. meleagridis* as well as subtypes of *C. meleagridis* in chickens in this study.

Sampling locations	No. positive/No. examined (%)	OR (95 CI)	No. positive *C. baileyi* (%)	No. positive *C. meleagridis* (%)/subtype (*n*)
Hotan1	5/22 (22.7)	11.8 (3.7–41.7)	5 (22.7)	0
Hotan2	0/16 (0)		0	0
Hotan3	0/45 (0)		0	0
Hotan4	0/14 (0)		0	0
Hotan5	6/13 (46.2)	34.3 (14.8–77.5)	5 (38.5)	1 (7.7)/IIIbA23G1R1 (1)
Hotan6	0/72 (0)		0	0
Karakax15	11/32 (34.4)	21.0 (17.0–51.8)	3 (9.4)	8 (25.0)/IIIbA25G1R1 (6)
Karakax16	9/29 (31.0)	18.0 (13.1–48.9)	3 (10.3)	6 (20.7)/IIIbA25G1R1 (4)
Lop7	5/38 (13.2)	6.0 (1.9–24.4)	0	5 (13.2)
Lop8	3/58 (5.2)	2.2 (0–11.0)	1 (1.7)	2 (3.4)
Lop9	0/22 (0)		0	0
Lop10	5/20 (25.0)	13.3 (4.2–45.8)	0	5 (25.0)/IIIbA25G1R1 (3)
Lop11	0/23 (0)		0	0
Minfeng12	21/46 (45.7)	33.6 (30.7–60.6)	11 (24.0)	10 (21.7)/IIIbA31G1R (3), IIIbA25G1R1 (7)
Minfeng13	0/20 (0)		0	0
Minfeng14	5/72 (6.9)	2.8 (0.9–13.0)	4 (5.6)	1 (1.4)/IIIbA31G1R (1)
Pishan17	0/34 (0)		0	0
Pishan18	1/41 (2.4)	1.0 (0–7.4)	1 (2.4)	0
Total	71/617 (11.5)	5.2 (0.9–14)	33 (5.3)	38 (6.2)/IIIbA23G1R1 (1), IIIbA25G1R1 (20), IIIbA31G1R (4)

**Table 2:** Infection and subtype distribution of *Cryptosporidium* species by age.

Age	No. positive/No. examined (%)	OR (95 CI)	No. positive *C. baileyi* (%)	No. positive *C. meleagridis* (%)/subtype (*n*)
<30 d	15/125 (12.0)	3.9 (6.2–17.8)	8 (6.4)	7 (5.6)/IIIbA23G1R1 (1), IIIbA25G1R1 (4)
30–60 d	48/331 (14.5)	4.9 (10.7–18.3)	20 (6.0)	28 (8.5)/IIIbA25G1R1 (16), IIIbA31G1R (4)
60–90 d	5/72 (6.9)	2.1 (0.9–13)	4 (5.6)	1 (1.4)
>90 d	3/89 (3.4)	1.0 (0–7.2)	1 (1.1)	2 (2.2)

### Genetic characterizations of *Cryptosporidium* at the *SSU* rRNA gene

From the 71 sequences of *Cryptosporidium* at the *SSU* rRNA gene, 38 (ON209452) were identical to *C. meleagridis* isolated from Parrot (MN410718), while 23 (ON209450) and 10 (ON209451) sequences were identical to *C. baileyi* isolated sequences from Chinese grosbeak (MN410723) and from silky fowl in China (KU744847), respectively. On the ten *Cryptosporidium* positive farms, *C. baileyi* was found on farms Hotan1 and Pishan18, and *C. meleagridis* was found on farms Lop7 and Lop10, while the remaining six farms were positive for both *C. meleagridis* and *C. baileyi* (Table 1)*.* No mixed infections were observed in any examined chickens.

The infection rates by *C. meleagridis* and *C. baileyi* were 7.7% (35/456) and 6.1% (28/456) in the <60 d group of Hotan black chickens, and 1.9% (3/161) and 3.1% (5/161) in the >60 d group, respectively. The infection rate by *C. meleagridis* was higher in the <60 d chickens than in the >60 d groups (Table 2). In contrast, the two age groups had no statistical difference in *C. baileyi* infection.

### Subtyping of *C. meleagridis* at the *gp60* gene

A total of 25 of the 38 *C. meleagridis* isolates were successfully PCR amplified at the *gp60* gene. Three subtypes of *C. meleagridis* were identified, including one known subtype (IIIbA23G1R1; *n* = 1) and two novel subtypes, named IIIbA25G1R1 (*n* = 20) and IIIbA31G1R1 (*n* = 4). Subtype IIIbA25G1R1 was only found on farms Lop10, Karakax15, and Karakax16, while subtypes IIIbA23G1R1 (*n* = 1) and IIIbA31G1R1 (*n* = 1) were only present on farms Hotan5 and Minfeng14, respectively. Farm Minfeng12 had both IIIbA31G1R and IIIbA25G1R1 subtypes (Table 1). All three subtypes of *C. meleagridis* were found only in <60 d groups of Hotan Black chickens (Table 2).

### Phylogenetic analysis of *C. meleagridis*

**Figure 2:**
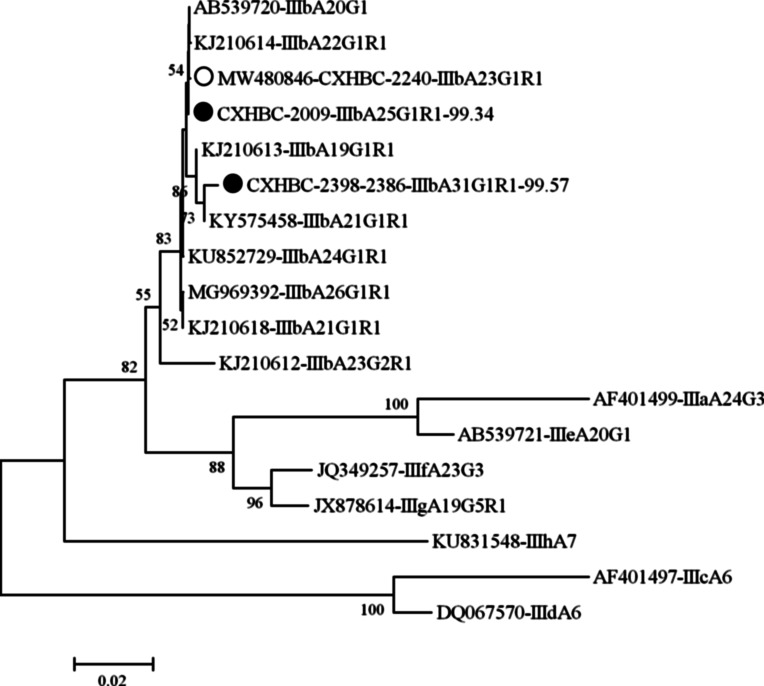
Phylogenetic tree based on the N-J analysis of gp60 sequences of *C. meleagridis*. Phylogenetic relationships of *C. meleagridis* subtypes identified in chicken here and parts of other known subtypes deposited in GenBank were inferred by a neighbor-joining analysis of gp60 sequences of *C. meleagridis* based on genetic distances by the Kimura two-parameter model. The numbers on the branches are percent bootstrapping values from 1000 replicates. The triangles filled in black and white indicate novel and known subtypes identified in this study, respectively.

All three subtypes of *C. meleagridis* obtained in this study clustered into subtype family IIIb, as expected (Fig. 2). The known subtype IIIbA23G1R1 and the novel one IIIbA25G1R1 clustered into one sub-branch together with IIIbA20G1 and IIIbA22G1R1, while subtype IIIbA31G1R1 clustered into a sub-branch together with IIIbA19G1R1 and IIIbA21G1R1.

## Discussion

Molecular epidemiological surveys of *Cryptosporidium* in chickens have been conducted in 12 countries with infection rates from 0.7% to 75.0% (Table 3) [1, 2, 4, 5, 7, 8, 11–14, 16, 17, 20, 21, 27–30, 32, 33]. This is the first study that revealed the presence of *Cryptosporidium* in chickens in Xinjiang, with an average infection rate of 11.5% which is higher than other studies from China (1.3–10.0%), Germany (7.0%), Iran (0.7–8.0%), Jordan (4.8%), Kenya (7.8%), Syria (9.9%), and Tunisia (4.5%) (Table 3). However, chickens from Algeria (15.3–34.0%), Bangladesh (15.7%), Brazil (12.6–25.6%), Côte d’Ivoire (16.1%), and Sweden (75.0%) had higher *Cryptosporidium* infection rates than those observed in the present study (Table 3). The differences in prevalence may be related to the sensitivity and specificity of the detection methods, the health status of hosts, territorial environment, overall sample size, feeding conditions, and drinking water sources, amongst others.

**Table 3:** Prevalence and species/genotype distribution of *Cryptosporidium* in chickens worldwide.

Country	No. positive/No. examined (%)	Species/genotypes of *Cryptosporidium* (*n*)	References
Algeria	31/90 (34.0)	*C. meleagridis* (26); *C. baileyi* (5)	[16]
	23/150 (15.3)	*C. baileyi* (12); *C. meleagridis* (11)	[17]
Bangladesh	31/197 (15.7)	*C. baileyi* (17); *C. meleagridis* (12); *C. parvum* (2).	[18]
Brazil	24/190 (12.6)	*C. baileyi* (18); *C. meleagridis* (1); *C. parvum* (4); *Cryptosporidium sp*. (1)	[19]
	20/130 (15.4)	*C. meleagridis* (15); *C. baileyi* (4); Mix (1)	[20]
	91/351 (25.6)^a^	*C. meleagridis* (57); *C. baileyi* (15); *C. parvum* (3); *C. genotype BrPR1* (3)	[21]
China	5/206 (2.4)	*C. baileyi* (5)	[10]
	179/2015 (8.9)	*C. meleagridis* (3); *C. baileyi* (176)	[11]
	38/385 (9.9)	*C. baileyi* (33); *C. meleagridis* (2); Avian genotype II (3)	[12]
	11/829 (1.3)	*C. baileyi* (5); *C. meleagridis* (2); *C. galli* (2); *C. xiao*i-like genotype (2)	[13]
	48/471 (10.2)	*C. meleagridis* (15); *C. baileyi* (33)	[14]
	132/1001 (13.2)	*C. meleagridis* (78); *C. baileyi* (48)	[22]
Côte d’Ivoire	5/31 (16.1)	*C. meleagridis* (4); *C. parvum* (1)	[23]
Germany	18/256 (7.0)	*C. parvum* (13); *C. baileyi* (2); *C. avian* genotypes VII, VIII, IX (one each)	[24]
Iran	7/1000 (0.7)	*C. baileyi* (7)	[25]
	8/100 (8.0)	*C. baileyi* (6); *C. parvum* (2)	[26]
Jordan	1/21 (4.8)	*C. baileyi* (1)	[27]
Kenya	25 (7.8)^a^	*C. andersoni* (2); *C. meleagridis* (4); *C. parvum* (3); *C. ubiquitum* (1)	[28]
Sweden	12/16 (75.0)^a^	*C. meleagridis* (6); *C. baileyi* (1); *C. galli* (3)	[9]
Syria	11/111 (9.9)	*C. baileyi* (9); Unknown genotype (2)^b^	[30]
Tunisia	9/200 (4.5)^a^	*C. meleagridis* (1)	[31]

a The numbers of species are not consistent with the numbers of positives in some countries because not all isolates were genotyped successfully for Brazil, Kenya, Sweden and Tunisia.

b Two samples showed unknown profiles in comparison to species.

In this study, the Hotan Black chickens aged 30–60 days had the highest infection rate by *Cryptosporidium* (14.5%), followed by those aged <30 days (12.0%), 60–90 days (6.9%) and >90 days (3.4%). In one previous study, chickens aged 61–90 days (17.8%) showed a significantly higher infection rate than chickens <30 d (8.3%), 31–60 days (12.0%) and >90 days (6.8%) of age, which was partially in agreement with the results of our study [22]. Usually, warming of chickens can be completely stopped at the age of >30 days, and they then leave the hotbed for flat-surfaced cages, living at their natural room temperature. At this age, the chickens’ immune system is not mature enough. Once the rearing environment is contaminated with *Cryptosporidium* oocysts, these chickens are easily infected with *Cryptosporidium*.

In this study, 38 *C. meleagridis* and 33 *C. baileyi* were identified, which was similar to a study where 26 *C. meleagridis* and five *C. baileyi* were identified in chickens in Algeria [1], and a study where 15 *C. meleagridis* and four *C. baileyi* were identified in chickens in Brazil [5]. Previously, 281 *Cryptosporidium* isolates were identified in chickens in China, with *C. baileyi* (*n* = 252) being the primary, widely distributed species, followed by *C. meleagridis* (*n* = 22), *C. ornithophilus* (synonym: *C. avian* genotype II) (*n* = 3), *C. galli* (*n* = 2), and *C. xiaoi*-like genotype (*n* = 2) [4, 11, 21, 32, 33]. In fact, all the studies from China except from Ezhou City, Hubei Province showed that both *C. meleagridis* and *C. baileyi* were present in chickens [4, 11, 21, 32, 33]. This shows that *C. baileyi* and *C. meleagridis* are two common species in chickens. However, the high proportion of *C. meleagridis* in chickens detected in the present study indicates that the constituent proportions of *C. baileyi* and *C. meleagridis* in the chickens were significantly different compared with other studies conducted in China, which may represent the geographical genetic distribution characteristics of *Cryptosporidium* in chickens in this area. This phenomenon may be caused by the different transmission routes of *Cryptosporidium* in southern Xinjiang due to its unique natural environment and geographical conditions.

*Cryptosporidium baileyi* is the most common species detected in chickens and has been reported in over 20 avian hosts [24]. This species was also detected in the stools of two immunodeficient patients, probably due to the disrupted immune system [6, 19]. In some birds, experimental cross-transmission of *C. baileyi* from naturally infected broilers to other hosts has been successful, but mice and goats did not become infected [24]. *Cryptosporidium meleagridis* has also been reported in a wide range of avian species, as well as in humans, dogs, deer, minks, cattle, and rodents [18, 24, 26]. In Peru and Thailand, *C. meleagridis* accounts for 10–20% of human *Cryptosporidium* infections, with a frequency similar to *C. parvum* infections [3, 25]. In China, *C. meleagridis* has been isolated from humans, broiler chickens, laying hens, quails, cattle, minks, and wastewater samples, suggesting *C. meleagridis* as a zoonotic disease agent with significance to human health [9]. Thus, it is necessary to avoid or reduce the occurrence, transmission, and re-infection of this pathogen among individuals within each farm by improving husbandry practices related to feeding and water.

To date, 28 subtypes have been identified in the subtype family IIIb, and 23 of these can infect humans, while the other five subtypes (IIIbA15G1, IIIbA21G1R1d, IIIbA22G1R1, IIIbA22G1R1a and IIIbA27G1) were found only in birds or water (Table S1). Therefore, the subtypes in family IIIb have potential zoonotic transmission. In this study, three subtypes (IIIbA23G1R1, IIIbA25G1R1, and IIIbA31G1R) were identified. However, subtypes IIIbA25G1R1 and IIIbA31G1R found here were not described previously, which may indicate geographical segregation characteristics in the distribution of *C. meleagridis* subtypes derived from chickens. More investigations will need to be done for source attribution of infection of the two subtypes and to understand their transmission dynamics in the future.

In conclusion, this study reports the first detection of *Cryptosporidium* in Hotan Black chicken with an 11.5% overall infection rate. *Cryptosporidium meleagridis* and *C. baileyi* were identified, with *C. meleagridis* being the predominated one, and two novel *C. meleagridis* subtypes were identified, named IIIbA25G1R1and IIIbA31G1R1. Therefore, future investigations on the genetic characteristics of regional distribution of avian *Cryptosporidium* should be conducted.

## Conflict of interest

The authors declare that they have no competing interests.

## Supplementary materials

The supplementary material is available at https://www.parasite-journal.org/10.1051/parasite/2022051/olm *Table S1*: Subtype of *C. meleagridis*.
