# Supplementary material for: Genetic characterization of Cryptosporidium spp. in Hotan Black Chickens in China reveals two novel subtypes of Cryptosporidium meleagridis
Source: Parasite. 2022 Nov 9;29:50. doi: 10.1051/parasite/2022051 (PMC9645226; doi:10.1051/parasite/2022051)
Supplement: Supplementary file 1 — Table S1: Subtype of C. meleagridis. [file parasite-29-50-s1.pdf]

Table S1: Subtype of *C. meleagridis.*

Family	Gene bank	Subtype	Host	Country	References
IIIa	MH062746	IIIaA22G3R1	Chicken	Brazil	da Cunha et al., 2018
	AF401499	IIIaA24G3R1a	Turkey	USA	Glaberman et al., 2001
	AF401500	IIIaA24G3R1b	Humans	Kenya	Glaberman et al.,2001
IIIb	KF733821	IIIbA13G1	Humans	Peru	Wang et al., 2014
	AF401501	IIIbA13G1R1	Humans	Peru	Glaberman et al.,2001
	KF733822	IIIbA15G1	Bird	Peru	Wang et al., 2014
	KJ210613	IIIbA19G1R1	Humans	Thailand	Stensvold et al., 2014
	AB539720	IIIbA20G1R1	Humans	Japan	Abe et al., 2010
	KJ210610	IIIbA21G1R1a	Humans	Sweden	Stensvold et al., 2014
	KJ210618	IIIbA21G1R1b	Humans	Thailand	7-Stensvold et al., 2014
	MT569393	IIIbA21G1R1d	Water	China	Jiang et al., 2020
	MG969391	IIIbA22G1R1	Chicken	China	Liao et al., 2018
	AF401502	IIIbA22G1R1a	Turkey	USA	Glaberman et al.,2001
	AB539719	IIIbA22G1R1b	Humans	Japan	Stensvold et al., 2014
	KJ210617	IIIbA22G1R1c	Humans	Thailand	Stensvold et al., 2014
	KJ210606	IIIbA23G1R1a	Humans	Indonesia	Stensvold et al., 2014
	KJ210609	IIIbA23G1R1b	Humans	Thailand	Stensvold et al., 2014
	KU852727	IIIbA23G1R1c	Humans	Malaysia	Unpublished
	KJ210612	IIIbA23G2R1	Humans	Indonesia	Stensvold et al., 2014
	MT952948	IIIbA23G4R1	Humans	Australia	Unpublished
	KF733818	IIIbA24G1	Humans	Peru	Wang et al., 2014
	KU852729	IIIbA24G1R1	Humans	China	Unpublished
	KF733816	IIIbA26G1	Humans	Peru	Wang et al., 2014
	MN746323	IIIbA26G1R1	Humans	Colombia	Galvan-Diaz et al., 2020
	AF401498	IIIbA26G1R1a	Human(HIV)	Kenya	Glaberman et al.,2001
	JQ029726	IIIbA26G1R1b	Human(HIV)	China	Wang et al., 2013
	KF733817	IIIbA27G1	Bird	Peru	Wang et al., 2014
	JF691562	IIIbA27G1R1	Humans	China	Wang et al., 2013
	JQ029725	IIIbA29G1R1	Humans	China	Wang et al., 2013
	KF733819	IIIbA30G1	Humans	Peru	Wang et al., 2014
	KF733820	IIIbA34G1	Humans	Peru	Wang et al., 2014
IIIc	KF733815	IIIcA6	Humans	Peru	Wang et al., 2014
	JF495149	IIIcA6R1	Humans	India	Stensvold et al., 2014
IIId	MW159785	IIIdA6	Humans	Malawi	Huston et al., 2021
	JF495148	IIIdA6R1	Humans	India	Stensvold et al., 2014
IIIe	AB539718	IIIeA16G2R1	Humans	Japan	Stensvold et al., 2014
	KP703169	IIIeA16G2R1b	Alectoris rufa	Czech Republic	Máca et al., 2015
	KU221105	IIIeA16G2R1c	Phasianus colchicus	Czech Republic	Máca et al., 2016
	KJ210608	IIIeA17G2R1	humans	Sweden	Stensvold et al., 2014
	MG516778	IIIeA18G2R1	Bird	Australia	Zahedi et al., 2018
	KJ210620	IIIeA19G2R1	humans	Uzbekistan	Stensvold et al., 2014
	AB539721	IIIeA20G1R1	cockatiel	Japan	Stensvold et al., 2014
	AB539717	IIIeA20G2R1	cockatiel	Japan	Stensvold et al., 2014
	KJ672091	IIIeA21G2R1	chicken	China	Unpublished

	JX416370	IIIeA22G2R1	dairy cattle	China	Zhang et al., 2013
	KJ210605	IIIeA25G2R1	Humans	India	Stensvold et al., 2014
	JF691563	IIIeA26G2R1	humans	China	Wang et al., 2013
IIIf	EU164813	IIIfA16G2R2	humans	Australia	Stensvold et al., 2014
IIIg	JX878613	IIIgA18G4R1	bird	Algeria	Baroudi et al., 2013
	JX878614	IIIgA19G5R1	bird	Algeria	Baroudi et al., 2013
	MH062749	IIIgA21G3R1a	Chicken	Brazil	da Cunha et al., 2018
	MH062750	IIIgA21G3R1b	Chicken	Brazil	da Cunha et al., 2018
	KU852730	IIIgA22G3R1	Humans	Nepal	Unpublished
	KY352483	IIIgA22G3R1	Gallus gallus	Algeria	Elkarim et al., 2017
	JQ349257	IIIgA23G3R1	humans	Spain	Stensvold et al., 2014
	JX878610	IIIgA24G2R1	bird	Algeria	Baroudi et al., 2013
	MH062752	IIIgA25G3R1	Chicken	Brazil	da Cunha et al., 2018
	OK562699	IIIgA25G3R1a	chicken	China	Unpublished
	JX878611	IIIgA26G3R1a	bird	Algeria	Baroudi et al., 2013
	JQ349258	IIIgA26G3R1b	Humans	UK	Stensvold et al., 2014
	KJ210619	IIIgA31G3R1	Humans	Sweden	Stensvold et al., 2014
IIIh	KU831548	IIIhA7	Humans	UK	Unpublished
	JQ349259	IIIhA24G3R1	Humans	UK	Abal-Fabeiro et al., 2013
IIIi	JQ349260	IIIiA23G3R1	Humans	UK	Abal-Fabeiroet al., 2013

References:

Abal-Fabeiro JL, Maside X, Bello X, Llovo J, Bartolomé C. Multilocus patterns of genetic variation across Cryptosporidium species suggest balancing selection at the gp60 locus. Mol Ecol. 2013 Sep;22(18):4723-32.

Abe N. Genotype analysis of Cryptosporidium meleagridis isolates from humans in Japan. Jpn J Infect Dis. 2010 May;63(3):214-5.

Baroudi D, Khelef D, Goucem R, Adjou KT, Adamu H, Zhang H, Xiao L. Common occurrence of zoonotic pathogen Cryptosporidium meleagridis in broiler chickens and turkeys in Algeria. Vet Parasitol. 2013 Sep 23;196(3-4):334-40.

da Cunha MJR, Cury MC, Santín M. Molecular characterization of Cryptosporidium spp. in poultry from Brazil. Res Vet Sci. 2018 Jun;118:331-335. doi: 10.1016/j.rvsc.2018.03.010. Epub 2018 Mar 21. PMID: 29605466.

Elkarim Laatamna A, Holubova N, Sak B, Kvac M. Cryptosporidium meleagridis and C. baileyi (Apicomplexa) in domestic and wild birds in Algeria. Folia Parasitol (Praha). 2017 Jun 13;64:2017.018. doi: 10.14411/fp.2017.018. PMID: 28620152.

Galvan-Diaz AL, Bedoya-Urrego K, Medina-Lozano A, Uran-Velasquez J, Alzate JF, Garcia-Montoya G. Common occurrence of Cryptosporidium hominis in children attending day-care centers in Medellin, Colombia. Parasitol Res. 2020 Sep;119(9):2935-2942. doi: 10.1007/s00436-020-06782-5. Epub 2020 Jun 27. PMID: 32594239.

Glaberman S, Sulaiman IM, Bern C, Limor J, Peng MM, Morgan U, Gilman R, Lal AA, Xiao L. A multilocus genotypic analysis of Cryptosporidium meleagridis. J Eukaryot Microbiol. 2001;Suppl:19S-22S.

Huston CD. The Clofazimine for Treatment of Cryptosporidiosis in HIV-Infected Adults (CRYPTOFAZ) and Lessons Learned for Anticryptosporidial Drug Development. Clin Infect Dis. 2021 Jul 15;73(2):192-194. doi: 10.1093/cid/ciaa425. PMID: 32277815; PMCID: PMC8427724.

Jex AR, Whipp M, Campbell BE, Cacciò SM, Stevens M, Hogg G, Gasser RB. A practical and cost-effective mutation scanning-based approach for investigating genetic variation in Cryptosporidium. Electrophoresis. 2007 Nov;28(21):3875-83.

Jiang W, Roellig DM, Li N, Wang L, Guo Y, Feng Y, Xiao L. Contribution of hospitals to the occurrence of enteric protists in urban wastewater. Parasitol Res. 2020 Sep;119(9):3033-3040. doi: 10.1007/s00436-020-06834-w. Epub 2020 Aug 4. PMID: 32748039.

Liao C, Wang T, Koehler AV, Fan Y, Hu M, Gasser RB. Molecular investigation of Cryptosporidium in farmed chickens in Hubei Province, China, identifies 'zoonotic' subtypes of C. meleagridis. Parasit Vectors. 2018 Aug 29;11(1):484. doi: 10.1186/s13071-018-3056-5. PMID: 30157928; PMCID: PMC6114272.

Máca O, Pavlásek I. Cryptosporidium infections of ring-necked pheasants (Phasianus colchicus) from an intensive artificial breeding programme in the Czech Republic. Parasitol Res. 2016 May;115(5):1915-22.

Máca O, Pavlásek I. First finding of spontaneous infections with Cryptosporidium baileyi and C. meleagridis in the red-legged partridge Alectoris rufa from an aviary in the Czech Republic. Vet Parasitol. 2015 Apr 30;209(3-4):164-8.

Muthusamy D, Rao SS, Ramani S, Monica B, Banerjee I, Abraham OC, Mathai DC, Primrose B, Muliyil J, Wanke CA, Ward HD, Kang G. Multilocus genotyping of Cryptosporidium sp. isolates from human immunodeficiency virus-infected individuals in South India. J Clin Microbiol. 2006 Feb;44(2):632-4.

Stensvold CR, Beser J, Axén C, Lebbad M. High applicability of a novel method for gp60-based subtyping of Cryptosporidium meleagridis. J Clin Microbiol. 2014 Jul;52(7):2311-9.

Wang L, Zhang H, Zhao X, Zhang L, Zhang G, Guo M, Liu L, Feng Y, Xiao L. Zoonotic Cryptosporidium species and Enterocytozoon bieneusi genotypes in HIV-positive patients on antiretroviral therapy. J Clin Microbiol. 2013 Feb;51(2):557-63.

Wang Y, Yang W, Cama V, Wang L, Cabrera L, Ortega Y, Bern C, Feng Y, Gilman R, Xiao L. Population genetics of Cryptosporidium meleagridis in humans and birds: evidence for cross-species transmission. Int J Parasitol. 2014 Jul;44(8):515-21.

Zahedi A, Monis P, Gofton AW, Oskam CL, Ball A, Bath A, Bartkow M, Robertson I, Ryan U. Cryptosporidium species and subtypes in animals inhabiting drinking water catchments in three states across Australia. Water Res. 2018 May 1;134:327-340. doi: 10.1016/j.watres.2018.02.005. Epub 2018 Feb 6. PMID: 29438893.

Zhang W, Wang R, Yang F, Zhang L, Cao J, Zhang X, Ling H, Liu A, Shen Y. Distribution and genetic characterizations of Cryptosporidium spp. in pre-weaned dairy calves in Northeastern China's Heilongjiang Province. PLoS One. 2013;8(1):e54857.
